# Age at menarche, age at natural menopause, and risk of rheumatoid arthritis — a Mendelian randomization study

**DOI:** 10.1186/s13075-021-02495-x

**Published:** 2021-04-09

**Authors:** Jingjing Zhu, Zheng Niu, Lars Alfredsson, Lars Klareskog, Leonid Padyukov, Xia Jiang

**Affiliations:** 1 The First Affiliated Hospital of Zhejiang Chinese Medical University, Hangzhou, China; 2 Department of Gynecology, Affiliated Hangzhou First People’s Hospital, Zhejiang University School of Medicine, Hangzhou, China; 3 Department of Clinical Neuroscience, Center for Molecular Medicine, Karolinska Institute, Tomtebodavägen 5, 17 177 Stockholm, Sweden; 4 Center for Molecular Medicine, Karolinska Institute, Stockholm, Sweden; 5 Program in Genetic Epidemiology and Statistical Genetics, Department of Epidemiology, Harvard T.H. Chan School of Public Health, Boston, USA; 6 West China School of Public Health and West China Fourth Hospital, Sichuan University, Chengdu, China

**Keywords:** Mendelian randomization, Rheumatoid arthritis, Age at menarche, Age at natural menopause, Age at first birth

## Abstract

**Background:**

Hormonal reproductive factors have been suggested to play an important role in the etiology of rheumatoid arthritis (RA), an autoimmune inflammatory disorder affecting primarily women. We conducted a two-sample Mendelian randomization (MR) study examining three relevant exposures, age at menarche (AAM), age at natural menopause (ANM), and age at first birth (AFB) with the risk of RA.

**Methods:**

We collected summary statistics from the hitherto largest GWAS conducted in AAM (*N* = 329,345), ANM (*N* = 69,360), AFB (*N* = 251,151), and RA (*N*_case_ = 14,361, *N*_control_ = 43,923), all of European ancestry. We constructed strong instruments using hundreds of exposure-associated genetic variants and estimated causal relationship through different MR approaches including an inverse-variance weighted method, an MR-Egger regression and a weighted median method. We conducted a multivariable MR to control for pleiotropic effect acting in particular through obesity and socioeconomic status. We also performed important sensitivity analyses to verify model assumptions.

**Results:**

We did not find any evidence in support for a causal association between genetically predicted reproductive factors and risk of RA (OR_per-SD increment in AAM_ = 1.06 [0.98–1.15]; OR_per-SD increment in ANM_ = 1.05 [0.98–1.11], OR _per-SD increment in AFB_ = 0.85 [0.65–1.10]). Results remained consistent after removing palindromic SNPs (OR_per-SD increment in AAM_ = 1.06 [0.97–1.15], OR_per-SD increment in ANM_ = 1.05 [0.98–1.13], OR_per-SD increment in AFB_ = 0.81 [0.61–1.07]) or excluding SNPs associated with potential confounding traits (OR_per-SD increment in AAM_ = 1.03 [0.94–1.12], OR_per-SD increment in ANM_ = 1.04 [0.95–1.14]). No outlying instrument was identified through the leave-one-out analysis.

**Conclusions:**

Our MR study does not convincingly support a casual effect of reproductive factors, as reflected by age at menarche, age at menopause, and age at first birth, on the development of RA. Despite the largely augmented set of instruments we used, these instruments only explained a modest proportion of phenotypic variance of exposures. Our knowledge regarding this topic is still insufficient and future studies with larger sample size should be designed to replicate or dispute our findings.

## Background

Rheumatoid arthritis (RA) is a chronic autoimmune inflammatory disorder affecting mainly women of reproductive age and often leads to disabling outcomes as well as shortened life expectancy if left untreated or not properly controlled. The sex difference in the prevalence of RA has been well documented where the disease strikes women more frequently and severe. For example, the incidence of RA has been estimated to be 4–5 times higher in women than in men below age 50 and twice higher during age 60–70 [1]. In addition, observational studies have suggested that in general, female RA patients do worse than male patients [2].

The reasons for this overrepresentation of women remain unclear but X-linked genetic factors and hormonal components are likely to be involved. Some women develop RA at transitional periods when sex hormones are shifting, for example after pregnancy and/or before menopause [3]. Medications that modulate hormone levels including long-term oral contraceptive use [4] and/or postmenopausal hormone therapy [5] have been found to be associated with a reduced risk of RA. These observations highlight a role of hormonal and reproductive factors in the disease etiology.

Several large-scale epidemiological studies have investigated the relationship between female reproductive factors and RA using three most readily available measures — age at menarche (AAM), age at natural menopause (ANM) and age at first birth (AFB) — yet results remain controversial. For example, the longitudinal Nurses’ Health Study enrolling 121,700 women during 1976–2002 identified an association of age at menarche ≤ 10 years with an increased risk of seropositive RA (RR 1.6, 95% CI 1.1–2.4) but not a significant association with risk of overall RA [6]. The study (NHS, 1976–2010; NHSII 1989–2011) also revealed that early age at natural menopause (≤ 44 years) was associated with an increased risk of seronegative RA (pooled HR 2.4, 95% CI 1.5–4.0) [7]. Data from the Swedish EIRA study, a population-based case-control study of female incident RA cases (2035 cases and 2911 controls, aged 18–70 years) showed an increased risk of ACPA (antibodies to citrullinated peptide antigens)-negative RA in those who were at a young age at first birth (< 23 years) (OR 2.5, 95% CI 1.5–4.1) compared to nulliparous women [8]. An analysis using cross-sectional data from 1892 participants in the Third National Health and Nutrition Examination Survey did not find any association between age at menarche or pregnancy history with RA after menopause [9]. These discrepancies are perhaps not surprising since conventional epidemiological studies generally rely on environmental information and results are likely to be influenced by measurement error, confounding, and reverse causality.

Hormonal reproductive factors including puberty and fertility are influenced by genetic, nutritional, socioeconomic, and environmental factors and can be highly heterogeneous among women. Nevertheless, the genetic regulations in AAM, ANM, and AFB have been recently highlighted by discoveries from large-scale genome-wide association studies (GWAS) leveraging millions of women of European ancestry. These results provide a valuable opportunity to utilize a novel statistical approach Mendelian randomization (MR) to make causal inference — an approach that uses genetic variants (single nucleotide polymorphisms, SNPs) as instrumental variables (IVs) to assess a causal effect of a risk factor on an outcome from observational data. Since SNPs are randomly assigned at conception and always precede disease onset, results from MR are less susceptible to confounding and reverse causation, which are the major limitations of conventional observational studies [10]. To the best of our knowledge, no MR analysis has been performed to examine a potential causal association between hormonal reproductive factors and development of RA, of which findings may help address patient concerns in topics of puberty, fertility, motherhood, and RA as well as improve our knowledge on the biological mechanisms underlying RA.

Therefore, we aim to conduct the first and also the largest two-sample MR on three female reproductive factors (AAM, ANM, and AFB) with the risk of RA. Genetic variants associated with each reproductive event were used as instrumental variable (IVs). IV-exposure associations were extracted from the recently published and also the largest GWAS(s) conducted in AAM (*N* = 329,345), ANM (*N* = 69,360), and AFB (*N* = 251,151) [11–13]. IV-outcome associations were extracted from the largest GWAS conducted in RA (*N*_RA_ = 14,361, *N*_control_ = 43,923) [14].

## Methods

We conducted current study applying a standard two-sample framework where IV-exposure associations and IV-outcome associations come from two sets of independent non-overlapping individuals. To reduce population stratification, we included only individuals of European ancestry.

### Exposure

Three reproductive exposures demonstrated by previous GWAS(s) as having polygenic components were involved. Age at menarche, a milestone in female pubertal development, varies markedly among females. The genetic regulation in AAM has been highlighted by a recent meta-GWAS incorporating 329,345 women collected by the ReproGen consortium, 23andMe, and UK Biobank and identified 389 independent AAM-associated signals spreading over 10 biological pathways [11]. Age at natural menopause poses a substantial impact on infertility and disease risk including breast cancer and cardiovascular events. The genetic architecture of ANM has been examined by a recent GWAS of 69,360 women identifying 54 independent signals located in 44 genomic regions, most of which were found to contain one or more DNA damage response pathway genes [12]. Reproductive behavior such as age at first birth is known to be partly driven by biological processes. A recent GWAS has examined the genetic architecture of reproductive tempo defined by AFB in 251,151 women and identified 10 AFB-associated loci [13].

In all three GWAS(s), independent signals (our IVs) were defined as the following: A list of index variants was first defined using a distance-based metric, by which any SNPs passing the two-tailed threshold of significance (*P* < 5 × 10^− 8^) within 1 Mb of another significant SNP were considered to be located in the same locus. This list of signals was then augmented using approximate conditional analysis in GCTA, using an LD reference panel from the UK Biobank study. Only secondary signals that were uncorrelated (*r*^2^ < 0.05) were included in the final list. All IVs passed quality control procedures under minor allele frequency > 0.001 and Hardy-Weinberg Equilibrium > 1 × 10^− 6^. IV-exposure associations were extracted from each GWAS [11–13].

### Outcome

IV-outcome associations were obtained from a meta-GWAS involving 18 participating cohorts totaling 14,361 RA cases and 43,923 controls of European ancestry. To the best of our knowledge, none of the participants in these 18 studies overlapped with participants in the exposure GWAS(s) [14].

### Statistical analysis

#### Mendelian randomization analysis

We applied several MR methods including an inverse-variance weighted approach (IVW) [15], a maximum likelihood-based method [16], an MR-Egger regression [17] and a weighted median approach [18].

Briefly, IVW represents the main conventional approach which only gives consistent estimates if all genetic variants in the analysis are valid instrumental variables. When the IVs are weak, IVW tends to underestimate the true variation of the estimate, while the likelihood method gives appropriately estimated confidence intervals. MR-Egger evaluates the directional pleiotropic effect of instrumental variables, of which the intercept term can be interpreted as an estimate of the average pleiotropy of genetic variations. The weighted median method is robust to outliers and provides consistent estimates even when 50% of the genetic variants are invalid IVs; and is considered as relatively more robust to horizontal pleiotropy.

#### Sensitivity analysis

A valid MR analysis is defined by three key model assumptions — the IVs are strongly associated with the risk factor of interest (relevance), share no common cause with the outcome (independence), and affect outcome solely through the exposure (exclusion restriction) [10]. Upon the satisfaction of all three assumptions, causal inferences between exposure(s) and outcome(s) can be made based on observational data.

We performed several important sensitivity analyses to verify MR model assumptions. For each index SNP, we searched for its potential association with confounding traits in GWAS catalog and conducted analysis excluding pleiotropic SNPs. Moreover, we used a robust adjusted profile score (MR-RAPS) approach which is robust to both systemic and idiosyncratic pleiotropy and performed excellently in all the numerical examples [19]. Educational attainment and obesity are two important confounders affecting both reproductive traits and risk of RA [20]. We further integrated GWAS summary statistics and additional IVs on education and BMI, and conducted an IVW-based multivariable MR (MVMR) to estimate the direct effect of reproductive factors controlling for the effect of BMI and education [21, 22]. Finally, we excluded one SNP at-a-time and performed IVW on the remaining SNPs to identify outlying IVs.

We calculated statistical power using the non-centrality parameter of the test statistic as suggested by Brion et al. (http://cnsgenomics.com/shiny/mRnd/). All analyses were conducted with packages “TwoSampleMR”, “MRInstruments”, and “Mendelian Randomization” in R v3.6.3.

#### Ethics/consent statement

Our study is a secondary analysis of existing, de-identified, summary-level GWAS data. Specific ethics and consent statement for each GWAS examined in this study can be found in the original GWAS publications.

## Results

Overall, we did not find convincing evidence in support for a causal relationship between the three hormonal related exposures and risk of RA. Specifically, genetically predicted AAM did not significantly influence the risk of RA using IVW approach (OR_per-SD increment in AAM_ [95% CI], 1.06 [0.98–1.15]). Estimates remained consistent across different methods (OR_per-SD increment in AAM_ [95% CI] for maximum likelihood 1.07 [1.00–1.14], for MR-Egger regression 1.11 [0.90–1.36], for weighted median 1.08 [0.97–1.21]). We did not observe apparent sign of pleiotropy (*P* for MR-Egger intercept = 0.69). Similarly, we did not find any compelling evidence supporting a casual association of genetically instrumented ANM with RA either using IVW (OR_per-SD increment in ANM_ [95% CI], 1.05 [0.98–1.11]) or MR-Egger regression (OR_per-SD increment in ANM_ [95% CI], 1.04 [0.90–1.20]). Null finding was identified using the weighted median approach (OR_per-SD increment in ANM_ [95% CI], 1.03 [0.98–1.08]). Consistently, for genetically predicted AFB, we did not observe any significant association with RA using IVW (OR_per-SD increment in AFB_ [95% CI], 0.85 [0.65–1.10]), MR-Egger regression (OR_per-SD increment in AFB_ [95% CI], 3.32 [0.36–30.81]), or weighted median approach (OR_per-SD increment in AFB_ [95% CI], 0.90 [0.73–1.10]). Indeed, for both ANM and AFB, significant results appeared using the maximum likelihood approach, yet this method is known to provide better power by neglecting horizontal pleiotropy and results were not supported by other methods. We did not observe apparent signs of horizontal pleiotropy (*P* for MR-Egger intercept = 0.92 for ANM and = 0.26 for AFB) (Table 1).

**Table 1:** Genetically predicted age at menarche, age at menopause and, age at first birth with the risk of rheumatoid arthritis. Results from primary Mendelian randomization analysis as well as sensitivity analyses based on a subset of instruments

Methods	#SNP	OR (95% CI)	*P*-value	#SNP	OR (95% CI)	*P*-value	#SNP	OR (95% CI)	*P*-value
	Full set			Remove palindromic SNPs			Remove confounding SNPs		
**Age at menarche**									
IVW	340	1.06 (0.98–1.15)	0.11	284	1.06 (0.97–1.15)	0.19	283	1.03 (0.94–1.12)	0.57
Maximum likelihood	340	1.07 (1.00–1.14)	0.06	284	1.06 (0.99–1.14)	0.12	283	1.03 (0.95–1.11)	0.50
MR-Egger	340	1.11 (0.90–1.36)	0.34	284	1.08 (0.85–1.37)	0.51	283	0.97 (0.72–1.30)	0.83
MR-Egger intercept			0.69			0.85			0.68
Weighted median	340	1.08 (0.97–1.21)	0.15	284	1.10 (0.97–1.25)	0.12	283	1.01 (0.90–1.14)	0.86
**Age at natural menopause**									
IVW	54	1.05 (0.98–1.11)	0.15	47	1.05 (0.98–1.13)	0.13	42	1.04 (0.95–1.14)	0.40
Maximum likelihood	54	1.05 (1.02–1.08)	4 × 10^−3^	47	1.06 (1.02–1.10)	9 × 10^−4^	42	1.05 (1.00–1.10)	0.05
MR-Egger	54	1.04 (0.90–1.20)	0.61	47	1.05 (0.90–1.23)	0.51	42	1.05 (0.74–1.48)	0.79
MR-Egger intercept			0.92			0.99			0.96
Weighted median	54	1.03 (0.98–1.08)	0.30	47	1.05 (1.00–1.11)	0.07	42	0.94 (0.88–1.01)	0.09
**Age at first birth**									
IVW	10	0.85 (0.65–1.10)	0.22	9	0.81 (0.61–1.07)	0.14	NA	NA	NA
Maximum likelihood	10	0.84 (0.73–0.97)	0.02	9	0.81 (0.70–0.94)	5 × 10^−3^	NA	NA	NA
MR-Egger	10	3.32 (0.36–30.81)	0.32	9	3.05 (0.33–28.49)	0.36	NA	NA	NA
MR-Egger intercept			0.26			0.28	NA	NA	NA
Weighted median	10	0.90 (0.73–1.10)	0.30	9	0.85 (0.69–1.05)	0.13	NA	NA	NA

*NA* none of the 10 age at first birth associated SNPs was found to be associated with other traits according to GWAS catalog, *IVW* inverse-variance weighted method, *OR* odds ratio, the risk of developing rheumatoid arthritis per-SD increment in age at menarche, age at natural menopause, or age at first birth

Results remained consistent after removing palindromic SNPs (OR [95% CI], 1.06 [0.97–1.14] for AAM; 1.05 [0.98–1.13] for ANM; 0.81 [0.61–1.07] for AFB). As shown in Supplementary Tables 1 and 2, the AAM and ANM IVs were also found to be associated with potential confounders while none of the 10 AFB-associated IVs was cited by the NHGRI-EBI Catalog (Supplementary Table 3). Using 283 AAM-associated IVs and 42 ANM-associated IVs excluding pleiotropic SNPs, we did not detect a causal effect of AAM or ANM on RA risk (OR [95% CI], 1.03 [0.94–1.12] for AAM, 1.04 [0.95–1.14] for ANM), corroborating our primary findings (Table 1).

To effectively control for pleiotropy, we next looked into the Robust Adjusted Profile Score (RAPS) approach which is robust to both systemic and idiosyncratic pleiotropy [19]. We performed MR-RAPS estimator and found that results remained largely consistent with our primary findings (Table 2).

**Table 2:** Genetically predicted age at menarche, age at menopause, and age at first birth with the risk of rheumatoid arthritis. A sensitivity analysis using MR-RAPS assuming over-dispersion

Methods of robust loss	OR (95% CI)	*P*-value
**Age at menarche**		
Huber method	1.07 (0.98–1.16)	0.13
Tukey method	1.07 (0.99–1.16)	0.08
**Age at natural menopause**		
Huber method	1.02 (0.97–1.07)	0.52
Tukey method	1.01 (0.97–1.05)	0.51
**Age at first birth**		
Huber method	0.90 (0.70–1.15)	0.41
Tukey method	0.89 (0.70–1.14)	0.37

Education and BMI are two modifiable risk factors, both of which play an important role in the etiology of RA and shape the reproductive exposures. We next conducted an IVW-based MVMR to estimate a direct effect of reproductive factors on RA accounting for the confounding effect from obesity and socioeconomic status. The results of MVMR remained consistent with our primary findings. The effect of AAM with RA did not alter substantially after adjusting for BMI (OR [95% CI], 0.97 [0.83–1.13]) or education (OR [95% CI], 1.07 [0.98–1.16]). Similarly, for ANM, we did not observe any significant effect with RA after adjusting for BMI (OR [95% CI], 1.06 [0.99–1.27]) or education (OR [95% CI], 1.04 [0.98–1.11]). For AFB, similar null effect was found after adjusting for BMI (OR [95% CI], 0.85 [0.57–1.24]) or education (OR [95% CI], 1.10 [0.58–2.11]) (Table 3).

**Table 3:** Genetically predicted age at menarche, age at menopause, and age at first birth with the risk of rheumatoid arthritis. Multivariable analysis adjusting for the effect of body mass index and year of education

Methods	#SNP	OR (95% CI)	*P*-value
**Age at menarche**			
Body mass index	140	0.97 (0.83–1.13)	0.12
Year of education	316	1.07 (0.98–1.16)	0.11
**Age at natural menopause**			
Body mass index	51	1.06 (0.99–1.27)	0.08
Year of education	54	1.04 (0.98–1.11)	0.18
**Age at first birth**			
Body mass index	10	0.85 (0.57–1.24)	0.40
Year of education	10	1.10 (0.58–2.11)	0.76

**Fig. 1:**
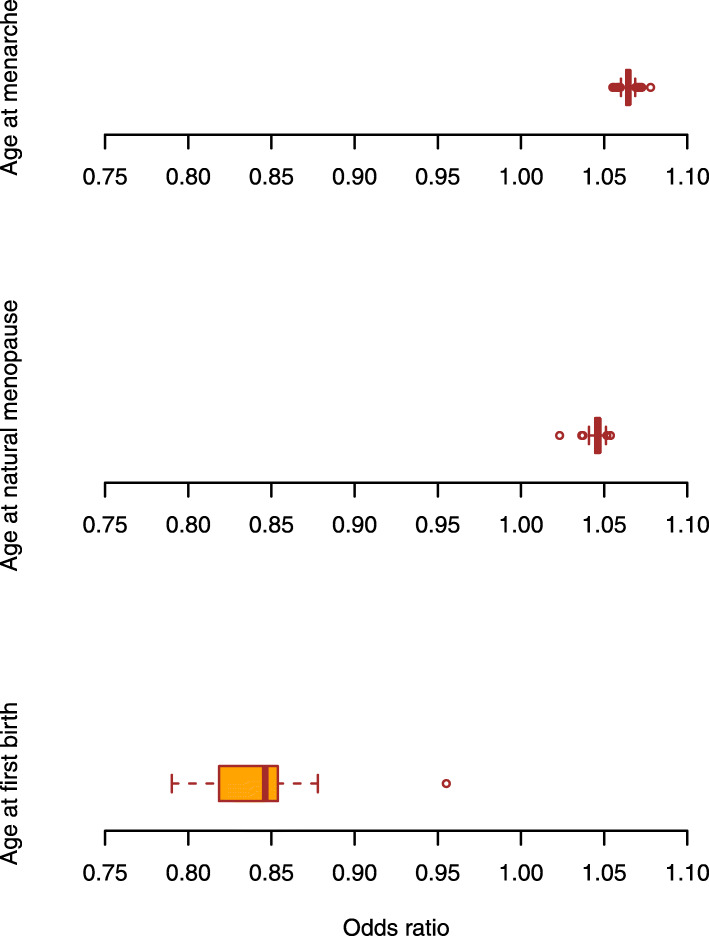
Sensitivity analysis leaving one SNP out at a time for the association between reproductive factors and RA risk. **a** The distribution of odds ratios from 389 leave-one-out analysis conducted for age at menarche and RA risk. **b** The distribution of odds ratios from 54 leave-one-out analysis conducted for age at menopause and RA risk. **c** The distribution of odds ratios from 10 leave-one-out analysis conducted for age at first birth and RA risk

In the leave-one-out analysis where we iteratively removed one SNP at a time and performed IVW using the remaining SNPs, we did not observe apparent outlying SNPs and the odds ratios were in accordance with our primary findings, aggregating closely around the expected value of estimation (Fig. 1).

Finally, we calculated the power of our analysis. As shown in Table 4, the sample size of the RA GWAS was 58,284 with 24.64% cases. According to the three exposure GWAS(s), 7.4% of phenotypic variance of AAM could be explained by the 389 index SNPs, 5.7% of ANM phenotypic variance could be explained by the 54 index SNPs, and 0.2% of AFB phenotypic variance could be explained by the 10 SNPs. Under current situation, for AAM, our study had 80% power to detect a causal effect of a 10.4% (i.e., ORs of 1.104) increase in RA risk. For ANM, the minimal detectable effect was 12% increase (i.e., ORs of 1.12). For AFB, the minimal detectable effect was 70% increase (i.e., ORs of 1.70). We presented a range of power estimations in Table 4.

**Table 4:** Power calculation of the current analysis

Exposure	Variance explained by index SNPs	Sample size (% cases)	Power to detect OR			
			1.05/0.95	1.10/0.91	1.15/0.87	1.20/0.83
Age at menarche	0.074	58,284 (24.64%)	28%	78%	98%	100%
Age at natural menopause	0.057		23%	67%	95%	100%
Age at first birth	0.002		6%	7%	10%	14%

## Discussion

In this study, we examined a putative causal relationship between three hormonal reproductive traits (AAM, ANM, and AFB) and an autoimmune inflammatory disease RA which affects mainly women. We capitalized on the summary statistics of the largest GWAS(s) conducted for these traits in European ancestry populations and constructed strong instruments using hundreds of SNPs associated with the exposures (F-statistic for AAM 67.6, for ANM 77.6, for AFB 50.3). We did not find convincing evidence in support for a causal effect of reproductive factors on RA using univariable MR analyses. Consistent null associations were identified by sensitivity analysis and multivariable MR analysis, demonstrating the robustness of our findings.

Current results from conventional epidemiological studies on this topic remain controversial, yet many studies point towards a positive association. For example, a study enrolling 121,700 female nurses found that age at menarche ≤ 10 years was associated with an increased risk of seropositive RA (RR 1.60, 95% CI 1.10–2.40) [6]. A community-based health survey including 30,447 subjects (18,326 women) between 1991 and 1996 found an association between early age at menopause (≤ 45 years) and subsequent development of RA (OR 2.42, 95% CI 1.32–4.45), which remained significant after adjusting for smoking, level of education and length of breastfeeding (OR 1.92, 95% CI 1.02–3.64) [23]. A prospective cohort study of 31,336 North America women reported similar findings (RR_menopause >51 vs. menopause <45_ 0.64, 95%CI 0.41–1.00) [24].

Our large-scale MR, however, did not identify a putative causal link between the three well-defined hormonal exposures and risk of RA. Several reasons underlie such a discrepancy. First of all, reproductive factors are highly complicated and heterogenous traits shaped by both genetic and environmental factors and genetics alone does not fully capture the phenotypic variance of these traits. For example, age at first birth is a human behavioral trait influenced largely by psychosocial, cultural, and financial factors rather than the genetics. Secondly, results from previous epidemiological studies are likely to be impaired by confounding factors. For example, obesity is an important confounder affecting both the exposure and the outcome. An MR study demonstrated that a 1-year delay in age at menarche reduced adult BMI by 0.38 kg/m^2^ (95% CI 0.25–0.51 kg/m^2^) [25]. Global adiposity is a robust causal risk factor for RA as demonstrated by our recently published MR [26]. It is likely that traditional epidemiological investigations did not adequately control for the confounding effects from obesity. The protective effect of education on RA has been reported by observational studies [27, 28]. An MR study identified that a 1-year later in age at menarche increased 0.14 years (53 days) of time spent in education [29]. We performed a MVMR to control for the effect of adiposity and education, and the negative results corroborating our main findings on a null association. Finally, it is also likely that the true causal effect of reproductive factors on RA is modest, which our study is underpowered to identify.

Biological mechanisms underlying hormonal factors and the development of RA remain unclear. The effect of sex hormones on the immune system and their interaction with environmental and genetic factors may partly explain the higher prevalence of RA observed among women. Estrogen is a complex modulator to the immune system exerting both a stimulatory and an inhibitory effect [30]. For example, estrogens at periovulatory to pregnancy levels stimulate B cells and the Th2 response and support the survival of auto-reactive T and B cell clones. On other hand, estrogens could inhibit cell-mediated responses such as the differentiation to Th17 cells [30–32]. A reduced risk of RA onset during pregnancy compared to an increased risk postpartum suggests a role the hormonal changes or the exposure to fetus paternal HLA in RA onset [33].

Our study has several strengths. To the best of our knowledge, no MR has been performed to assess the relationship between reproductive factors and RA. We incorporated three different reproductive traits (age at menarche, age at natural menopause, and age at first birth) reflecting the length of reproductive period and complementing each other well. Moreover, we conducted important sensitivity analyses to verify MR model assumptions. We selected the most significant independent SNPs identified by the largest GWAS, so all were robustly associated with exposure of interest, guaranteeing “relevance” assumption. We excluded SNPs associated with potential confounders on the exposure-outcome relationship to satisfy “exclusion restriction” assumption. The consistent results observed across different approaches, further lend support to our findings.

We have to acknowledge several limitations. Firstly, our analysis was performed using the European populations which restricted its generalizability. Secondly, the genetic instruments of three exposures (AAM, ANM, and AFB) we used as proxies for hormonal reproductive characteristics captured only a modest proportion of phenotypic variance. Reproductive factors are complex traits influenced by different components such as genetic, environmental, and socioeconomic factors as well as their complex interactions. The design of our study disables us to take into account environmental impacts. Thirdly, the association between genetically predicted age at each of the reproductive events and risk of RA was evaluated fitting the exposure as a continuous variable — we can still not exclude a non-linear effect which was not captured by our study with the current availability of data. Future work on such topics may be focused on categorized age of reproductive events. Fourthly, our study was conducted using overall RA (a majority of which are seropositive RA, > 85%) without specifying disease subsets characterized by the presence/absence of antibodies to citrullinated peptides or rheumatic factors. It is possible that hormonal factors influence different RA subsets via a distinct way. It is also likely that other factors such as hormone use and health conditions confound our results, in addition to the only two confounders (obesity and education) considered in the current study. However, it is difficult to control for the effect of hormone therapy due to limited availability of genetic data underlying this trait. Finally, power calculations showed that potential weak effects were difficult to be detected in our analysis.

## Conclusions

In summary, using both univariable and multivariable Mendelian randomization approaches, we could not provide evidence supporting a casual effect of reproductive factors as reflected by age at menarche, age at menopause, or age at first birth in the development of RA. Our result is not so surprising considering the relatively weak genetic instruments and power. The findings represent a preliminary but important step towards the identification of causal associations between female hormonal reproductive factors and a female disease RA. As some hormonal factors are potentially modifiable, understanding their precise role is essential for future preventive interventions focusing on women at high risk. Our knowledge regarding this topic is still insufficient and future studies with larger sample size and better power should be designed to increase our knowledge in this field.

## Supplementary Information

**Additional file 1: Supplementary Table 1.** The characteristic of age at menarche associated index SNPs, their effect sizes with exposure and outcome, as well as their associations with potential confounders. **Supplementary Table 2.** The characteristic of age at natural menopause associated index SNPs, their effect sizes with exposure and outcome, as well as their associations with potential confounders. **Supplementary Table 3.** The characteristic of age at first birth associated index SNPs, their effect sizes with exposure and outcome.

## Data Availability

The datasets used and/or analyzed during the current study are available from the corresponding author on reasonable request.

## Abbreviations

RA: Rheumatoid arthritis
AAM: Age at menarche
ANM: Age at natural menopause
AFB: Age at first birth
ACPA: Antibodies to citrullinated peptide antigens
MR: Mendelian randomization
GWAS: Genome-wide association studies
SNPs: Single nucleotide polymorphisms
IV: Instrumental variable
IVW: Inverse-variance weighted method
BMI: Body mass index
MVMR: Multivariable Mendelian randomization
