# Supplementary material for: Age at menarche, age at natural menopause, and risk of rheumatoid arthritis — a Mendelian randomization study
Source: Arthritis Res Ther. 2021 Apr 9;23:108. doi: 10.1186/s13075-021-02495-x (PMC8034136; doi:10.1186/s13075-021-02495-x)
Supplement: Supplementary file 1 — Additional file 1: Supplementary Table 1. The characteristic of age at menarche associated index SNPs, their effect sizes with exposure and outcome, as well as their associations with potential confounders. Supplementary Table 2. The characteristic of age at natural menopause associated index SNPs, their effect sizes with exposure and outcome, as well as their associations with potential confounders. Supplementary Table 3. The characteristic of age at first birth associated index SNPs, their effect sizes with exposure and outcome. [file 13075_2021_2495_MOESM1_ESM.docx]

| **Supplementary Table 1. The characteristic of age at menarche associated index SNPs, their effect sizes with exposure and outcome, as well as their associations with potential confounders.** | | | | | | | | | | |
| --- | --- | --- | --- | --- | --- | --- | --- | --- | --- | --- |
| SNP | Chr | Position | Allele frequency | A1 | A2 | Exposure | | Outcome | | Confounder |
|  |  |  |  |  |  | beta | se | beta | se |  |
| rs6678140 | 1 | 8436802 | 0.673 | T | C | -0.027 | 0.004 | -0.018 | 0.016 |  |
| rs12125335 | 1 | 21385436 | 0.860 | T | C | -0.050 | 0.006 | 0.109 | 0.035 | Smoking initiation, subjective well-being |
| rs141847393 | 1 | 27212209 | 0.918 | T | C | 0.040 | 0.007 | 0.078 | 0.037 |  |
| rs360495 | 1 | 33228197 | 0.087 | A | T | 0.043 | 0.007 | 0.009 | 0.038 |  |
| rs4970598 | 1 | 38940215 | 0.959 | T | C | 0.062 | 0.011 | 0.097 | 0.066 |  |
| rs11209331 | 1 | 41456689 | 0.571 | T | C | 0.024 | 0.004 | 0.015 | 0.017 |  |
| rs11210871 | 1 | 44029353 | 0.298 | C | G | 0.040 | 0.004 | 0.043 | 0.017 | Intelligence (MTAG) |
| rs643428 | 1 | 54728858 | 0.593 | T | C | -0.022 | 0.004 | -0.016 | 0.021 |  |
| rs7516763 | 1 | 65972550 | 0.469 | A | C | 0.023 | 0.004 | -0.005 | 0.016 |  |
| rs11209943 | 1 | 72750500 | 0.391 | A | G | 0.038 | 0.004 | 0.014 | 0.020 | General cognitive ability |
| rs1040070 | 1 | 74977870 | 0.565 | C | G | 0.050 | 0.004 | -0.027 | 0.020 |  |
| rs10782777 | 1 | 82562929 | 0.640 | A | G | -0.025 | 0.004 | -0.027 | 0.020 |  |
| rs11165924 | 1 | 98375448 | 0.677 | A | G | 0.031 | 0.004 | -0.016 | 0.023 |  |
| rs4561063 | 1 | 102520898 | 0.461 | T | G | 0.031 | 0.004 | -0.049 | 0.020 |  |
| rs61817552 | 1 | 150423577 | 0.211 | A | G | -0.029 | 0.005 | 0.003 | 0.024 |  |
| rs6661100 | 1 | 150758727 | 0.092 | T | C | 0.047 | 0.007 | -0.070 | 0.035 |  |
| rs4845364 | 1 | 154141908 | 0.492 | A | G | 0.022 | 0.004 | 0.033 | 0.019 |  |
| rs9427116 | 1 | 154631123 | 0.488 | T | C | 0.021 | 0.004 | 0.009 | 0.020 | Blood protein levels |
| rs2343507 | 1 | 162895515 | 0.588 | A | C | 0.023 | 0.004 | -0.027 | 0.017 |  |
| rs2661339 | 1 | 163018934 | 0.054 | T | G | 0.053 | 0.009 | 0.042 | 0.045 |  |
| rs157877 | 1 | 165398744 | 0.127 | A | G | -0.084 | 0.006 | 0.039 | 0.029 | Menopause (age at onset) |
| rs7542538 | 1 | 165426193 | 0.813 | T | C | 0.029 | 0.005 | -0.012 | 0.020 |  |
| rs506589 | 1 | 177894287 | 0.803 | T | C | 0.070 | 0.005 | 0.035 | 0.022 | Obese vs. thin |
| rs61828391 | 1 | 179732142 | 0.126 | A | G | -0.032 | 0.006 | 0.014 | 0.029 |  |
| rs11240695 | 1 | 204158132 | 0.251 | A | C | -0.033 | 0.004 | 0.042 | 0.022 |  |
| rs4951261 | 1 | 205717823 | 0.614 | A | C | 0.027 | 0.004 | 0.016 | 0.016 | Atrial fibrillation |
| rs12040029 | 1 | 213451958 | 0.128 | T | C | -0.039 | 0.006 | -0.027 | 0.031 |  |
| rs62104180 | 2 | 466003 | 0.051 | A | G | 0.113 | 0.010 | -0.090 | 0.093 | Body fat percentage, alcohol comsumption |
| rs7576624 | 2 | 625029 | 0.826 | T | C | -0.074 | 0.005 | -0.019 | 0.026 |  |
| rs7587651 | 2 | 10368606 | 0.373 | T | C | -0.024 | 0.004 | 0.016 | 0.021 |  |
| rs150821390 | 2 | 24106445 | 0.028 | T | C | 0.072 | 0.012 | 0.108 | 0.059 |  |
| rs72787511 | 2 | 32816089 | 0.034 | C | G | 0.065 | 0.011 | 0.028 | 0.055 |  |
| rs10175423 | 2 | 42970161 | 0.297 | T | C | -0.025 | 0.004 | 0.020 | 0.022 |  |
| rs17390720 | 2 | 44952254 | 0.735 | C | G | 0.026 | 0.004 | -0.020 | 0.022 |  |
| rs1025128 | 2 | 60175475 | 0.568 | C | G | -0.022 | 0.004 | -0.023 | 0.016 | Carpal tunnel syndrome |
| rs10205969 | 2 | 61367664 | 0.145 | T | C | -0.039 | 0.005 | -0.060 | 0.022 |  |
| rs12467441 | 2 | 61685826 | 0.874 | T | C | -0.041 | 0.006 | -0.048 | 0.023 |  |
| rs2723065 | 2 | 65279414 | 0.613 | A | G | -0.025 | 0.004 | -0.002 | 0.016 |  |
| rs2312205 | 2 | 69704941 | 0.822 | A | G | 0.030 | 0.005 | -0.016 | 0.025 |  |
| rs34437050 | 2 | 73535526 | 0.012 | A | G | 0.241 | 0.020 | 0.045 | 0.113 |  |
| rs2679894 | 2 | 105870779 | 0.437 | A | G | 0.051 | 0.004 | 0.060 | 0.023 | Menopause (age at onset) |
| rs2558101 | 2 | 137613322 | 0.720 | A | G | -0.024 | 0.004 | -0.002 | 0.021 |  |
| rs35935052 | 2 | 142302503 | 0.148 | T | G | 0.044 | 0.005 | 0.003 | 0.021 | Body mass index |
| rs6434162 | 2 | 153556801 | 0.824 | A | G | -0.036 | 0.005 | -0.016 | 0.026 |  |
| rs142058842 | 2 | 156621725 | 0.830 | C | G | -0.068 | 0.005 | -0.006 | 0.027 | Age at voice drop |
| rs145438026 | 2 | 157228255 | 0.064 | T | C | -0.070 | 0.008 | 0.049 | 0.041 |  |
| rs2271758 | 2 | 172701157 | 0.411 | T | G | -0.021 | 0.004 | -0.009 | 0.016 |  |
| rs842567 | 2 | 184291116 | 0.794 | A | C | -0.034 | 0.005 | 0.037 | 0.025 |  |
| rs10931831 | 2 | 199621641 | 0.356 | T | C | -0.053 | 0.004 | -0.025 | 0.016 | Menopause (age at onset) |
| rs13023912 | 2 | 199756278 | 0.652 | A | G | -0.051 | 0.004 | -0.007 | 0.016 |  |
| rs16841867 | 2 | 203168235 | 0.885 | C | G | 0.046 | 0.006 | -0.037 | 0.030 |  |
| rs184033703 | 2 | 206956138 | 0.058 | A | G | -0.048 | 0.009 | -0.048 | 0.044 | Chronotype |
| rs6735626 | 2 | 213403972 | 0.437 | A | G | 0.022 | 0.004 | -0.011 | 0.020 |  |
| rs73820560 | 3 | 1906245 | 0.856 | A | C | -0.032 | 0.006 | 0.030 | 0.030 |  |
| rs9867904 | 3 | 18442437 | 0.387 | C | G | -0.028 | 0.004 | 0.024 | 0.020 |  |
| rs73035994 | 3 | 24206463 | 0.972 | T | C | -0.091 | 0.012 | 0.035 | 0.058 |  |
| rs1984870 | 3 | 24715135 | 0.473 | T | G | 0.042 | 0.004 | -0.030 | 0.020 |  |
| rs77955256 | 3 | 44883523 | 0.106 | A | T | -0.036 | 0.006 | 0.017 | 0.032 |  |
| rs6803264 | 3 | 49254427 | 0.230 | T | C | 0.030 | 0.005 | 0.011 | 0.018 |  |
| rs115435316 | 3 | 49568181 | 0.033 | A | G | 0.115 | 0.011 | -0.002 | 0.044 |  |
| rs6445624 | 3 | 51358019 | 0.147 | A | G | 0.042 | 0.006 | -0.009 | 0.022 |  |
| rs10933 | 3 | 52719816 | 0.442 | T | C | -0.024 | 0.004 | -0.032 | 0.019 | Waist-hip ratio |
| rs7431217 | 3 | 68595634 | 0.412 | T | C | 0.023 | 0.004 | 0.013 | 0.020 |  |
| rs7426534 | 3 | 84462073 | 0.709 | A | G | -0.024 | 0.004 | 0.004 | 0.021 |  |
| rs9758500 | 3 | 86910329 | 0.375 | A | G | -0.046 | 0.004 | 0.004 | 0.020 |  |
| rs4859001 | 3 | 88221517 | 0.148 | T | C | 0.045 | 0.006 | 0.025 | 0.028 |  |
| rs709488 | 3 | 107700952 | 0.534 | A | C | -0.022 | 0.004 | -0.021 | 0.019 |  |
| rs9834893 | 3 | 114574749 | 0.929 | C | G | -0.051 | 0.007 | 0.019 | 0.038 |  |
| rs10934420 | 3 | 117552111 | 0.505 | T | C | -0.055 | 0.004 | -0.001 | 0.019 | Menopause (age at onset) |
| rs2461794 | 3 | 127870060 | 0.276 | A | G | 0.034 | 0.004 | 0.034 | 0.022 |  |
| rs6439371 | 3 | 132610752 | 0.648 | A | G | -0.030 | 0.004 | 0.025 | 0.021 |  |
| rs6439713 | 3 | 137128815 | 0.315 | A | C | 0.026 | 0.004 | -0.003 | 0.021 |  |
| rs11711674 | 3 | 156532953 | 0.571 | T | C | 0.022 | 0.004 | 0.000 | 0.016 |  |
| rs13322435 | 3 | 156795468 | 0.581 | A | G | 0.036 | 0.004 | 0.026 | 0.021 | Birth weight |
| rs582780 | 3 | 172121443 | 0.582 | A | G | 0.027 | 0.004 | -0.011 | 0.020 |  |
| rs7649124 | 3 | 184030827 | 0.757 | C | G | 0.033 | 0.005 | 0.019 | 0.023 |  |
| rs2300922 | 3 | 185651469 | 0.414 | T | C | 0.043 | 0.004 | -0.029 | 0.020 |  |
| rs2108753 | 4 | 3266860 | 0.565 | T | C | 0.028 | 0.004 | -0.026 | 0.020 |  |
| rs4340786 | 4 | 28746246 | 0.741 | A | T | 0.037 | 0.004 | 0.002 | 0.022 |  |
| rs4588499 | 4 | 45910674 | 0.494 | A | G | -0.024 | 0.004 | -0.016 | 0.020 |  |
| rs3113862 | 4 | 95143122 | 0.599 | A | G | -0.037 | 0.004 | -0.026 | 0.020 |  |
| rs55784701 | 4 | 104247262 | 0.225 | T | C | 0.026 | 0.005 | 0.037 | 0.024 |  |
| rs3733632 | 4 | 104640935 | 0.844 | A | G | -0.054 | 0.005 | -0.028 | 0.027 |  |
| rs17035311 | 4 | 106066293 | 0.854 | A | C | 0.036 | 0.005 | -0.005 | 0.022 |  |
| rs62316795 | 4 | 132621869 | 0.193 | A | C | 0.035 | 0.005 | -0.001 | 0.029 |  |
| rs13120031 | 4 | 177465182 | 0.324 | T | C | 0.027 | 0.004 | -0.020 | 0.017 |  |
| rs10521021 | 5 | 35030311 | 0.658 | T | G | -0.024 | 0.004 | 0.029 | 0.021 |  |
| rs62361685 | 5 | 41994067 | 0.942 | T | C | 0.054 | 0.009 | -0.026 | 0.046 |  |
| rs7712046 | 5 | 43134968 | 0.696 | T | C | -0.033 | 0.004 | -0.009 | 0.022 |  |
| rs813301 | 5 | 52909927 | 0.626 | T | C | 0.027 | 0.004 | 0.006 | 0.020 |  |
| rs256350 | 5 | 59140876 | 0.726 | T | C | -0.024 | 0.004 | 0.000 | 0.022 |  |
| rs80170948 | 5 | 64020316 | 0.956 | T | G | -0.069 | 0.011 | 0.050 | 0.058 | Cognitive performance |
| rs13173441 | 5 | 77048448 | 0.879 | T | C | 0.033 | 0.006 | 0.000 | 0.030 |  |
| rs17085593 | 5 | 95630705 | 0.684 | C | G | 0.025 | 0.004 | -0.008 | 0.021 |  |
| rs2916578 | 5 | 107316227 | 0.585 | A | G | -0.025 | 0.004 | 0.012 | 0.020 |  |
| rs654354 | 5 | 110503301 | 0.383 | A | T | -0.023 | 0.004 | 0.009 | 0.020 |  |
| rs247520 | 5 | 110876057 | 0.765 | T | C | 0.036 | 0.005 | -0.032 | 0.023 |  |
| rs1566385 | 5 | 111130474 | 0.942 | A | G | 0.060 | 0.008 | -0.040 | 0.043 |  |
| rs62379978 | 5 | 133915969 | 0.842 | T | G | -0.064 | 0.005 | 0.059 | 0.029 |  |
| rs3815212 | 5 | 137761555 | 0.783 | T | C | 0.034 | 0.005 | 0.011 | 0.024 |  |
| rs6878910 | 5 | 138281261 | 0.153 | A | G | 0.038 | 0.006 | 0.008 | 0.032 |  |
| rs975642 | 5 | 139384490 | 0.494 | T | C | -0.025 | 0.004 | 0.008 | 0.020 |  |
| rs71592143 | 5 | 141656062 | 0.539 | A | T | -0.029 | 0.005 | -0.020 | 0.022 |  |
| rs1428120 | 5 | 153541904 | 0.572 | T | G | 0.025 | 0.004 | 0.006 | 0.020 |  |
| rs437836 | 5 | 156715068 | 0.167 | T | C | 0.035 | 0.005 | -0.021 | 0.026 |  |
| rs9647570 | 5 | 167370263 | 0.854 | T | G | -0.036 | 0.006 | -0.009 | 0.029 |  |
| rs2546959 | 5 | 167404411 | 0.827 | T | G | 0.032 | 0.005 | 0.003 | 0.027 |  |
| rs4976623 | 5 | 167947996 | 0.189 | C | G | 0.030 | 0.005 | 0.010 | 0.020 |  |
| rs6864818 | 5 | 168734867 | 0.211 | T | C | 0.036 | 0.005 | -0.013 | 0.024 |  |
| rs4701140 | 5 | 179034260 | 0.512 | A | G | 0.024 | 0.004 | -0.004 | 0.021 |  |
| rs2770957 | 5 | 180656734 | 0.777 | C | G | 0.032 | 0.005 | -0.006 | 0.024 |  |
| rs446745 | 6 | 14918298 | 0.241 | T | C | -0.026 | 0.005 | 0.023 | 0.024 |  |
| rs6927679 | 6 | 18559687 | 0.718 | T | C | 0.027 | 0.004 | -0.006 | 0.022 |  |
| rs1539310 | 6 | 22562485 | 0.757 | A | G | 0.024 | 0.005 | -0.004 | 0.020 |  |
| rs12663002 | 6 | 28441634 | 0.129 | T | C | 0.043 | 0.006 | 0.097 | 0.022 |  |
| rs62391851 | 6 | 29740548 | 0.059 | A | G | -0.060 | 0.009 | 0.006 | 0.033 |  |
| rs9349203 | 6 | 41893323 | 0.546 | A | G | -0.040 | 0.004 | -0.012 | 0.019 |  |
| rs222440 | 6 | 52946320 | 0.186 | T | C | -0.033 | 0.005 | 0.002 | 0.026 |  |
| rs9474996 | 6 | 54640512 | 0.555 | A | T | -0.034 | 0.004 | 0.007 | 0.020 |  |
| rs9382676 | 6 | 56859084 | 0.777 | T | C | 0.037 | 0.005 | -0.024 | 0.023 |  |
| rs7753896 | 6 | 76347020 | 0.368 | A | G | 0.031 | 0.004 | 0.005 | 0.020 |  |
| rs7757654 | 6 | 77173780 | 0.296 | T | C | -0.031 | 0.004 | 0.033 | 0.021 |  |
| rs1414186 | 6 | 77713859 | 0.205 | T | G | -0.043 | 0.005 | -0.017 | 0.021 |  |
| rs11756746 | 6 | 84286477 | 0.243 | A | G | 0.025 | 0.005 | 0.017 | 0.023 |  |
| rs6931884 | 6 | 100158873 | 0.129 | T | C | 0.059 | 0.006 | 0.040 | 0.029 |  |
| rs9403051 | 6 | 100194846 | 0.570 | A | G | 0.037 | 0.004 | 0.020 | 0.020 |  |
| rs13199764 | 6 | 100744134 | 0.776 | T | C | 0.039 | 0.005 | -0.025 | 0.023 |  |
| rs12200565 | 6 | 100983589 | 0.539 | T | C | 0.032 | 0.004 | 0.045 | 0.020 |  |
| rs395962 | 6 | 105397418 | 0.316 | T | G | 0.127 | 0.004 | 0.025 | 0.018 | Waist circumference, BMI, height |
| rs6911407 | 6 | 108867031 | 0.376 | A | C | 0.029 | 0.004 | 0.002 | 0.020 |  |
| rs4327718 | 6 | 128364709 | 0.828 | A | G | -0.033 | 0.005 | -0.013 | 0.026 |  |
| rs78928932 | 6 | 136228617 | 0.931 | T | C | -0.055 | 0.009 | -0.048 | 0.041 |  |
| rs117530880 | 6 | 146687748 | 0.970 | T | G | -0.066 | 0.012 | 0.036 | 0.060 |  |
| rs6911527 | 6 | 148285329 | 0.228 | T | C | 0.027 | 0.005 | -0.002 | 0.023 |  |
| rs6933660 | 6 | 151803754 | 0.317 | A | C | -0.034 | 0.004 | -0.022 | 0.021 |  |
| rs910425 | 6 | 170652191 | 0.448 | A | G | -0.022 | 0.004 | -0.025 | 0.016 |  |
| rs10268051 | 7 | 27763590 | 0.777 | A | C | 0.025 | 0.005 | -0.006 | 0.019 |  |
| rs17171852 | 7 | 41392815 | 0.808 | A | C | -0.038 | 0.005 | 0.013 | 0.025 |  |
| rs1079866 | 7 | 41470093 | 0.863 | C | G | -0.071 | 0.006 | 0.016 | 0.029 |  |
| rs1470750 | 7 | 50576648 | 0.592 | C | G | -0.022 | 0.004 | 0.010 | 0.016 |  |
| rs2267812 | 7 | 74138121 | 0.795 | A | C | 0.042 | 0.005 | -0.053 | 0.021 |  |
| rs187760798 | 7 | 75142551 | 0.884 | T | C | 0.058 | 0.007 | 0.023 | 0.030 |  |
| rs1030015 | 7 | 78139581 | 0.522 | T | G | -0.021 | 0.004 | 0.000 | 0.016 | Smoking initiation (ever regular vs never regular) |
| rs149226155 | 7 | 93215658 | 0.347 | A | G | -0.024 | 0.004 | 0.009 | 0.024 |  |
| rs15671 | 7 | 94186064 | 0.421 | A | C | -0.023 | 0.004 | -0.019 | 0.020 |  |
| rs999885 | 7 | 99701176 | 0.516 | A | G | 0.024 | 0.004 | -0.028 | 0.019 |  |
| rs1456031 | 7 | 114296102 | 0.465 | T | C | 0.022 | 0.004 | -0.037 | 0.020 | Age of smoking initiation (MTAG) |
| rs10237306 | 7 | 121955981 | 0.390 | T | G | 0.030 | 0.004 | -0.010 | 0.020 |  |
| rs11767400 | 7 | 122160742 | 0.296 | A | C | 0.029 | 0.004 | -0.051 | 0.022 |  |
| rs11556924 | 7 | 129663496 | 0.370 | T | C | 0.023 | 0.004 | 0.015 | 0.025 | Cardiovascular disease, coronary artery disease |
| rs17563472 | 7 | 130409054 | 0.965 | T | C | -0.061 | 0.011 | 0.030 | 0.053 |  |
| rs12707076 | 7 | 132729814 | 0.384 | C | G | 0.027 | 0.004 | 0.050 | 0.020 |  |
| rs13233916 | 7 | 138874416 | 0.907 | C | G | -0.050 | 0.008 | -0.037 | 0.039 |  |
| rs7004265 | 8 | 1523903 | 0.477 | T | C | 0.023 | 0.004 | -0.007 | 0.020 |  |
| rs2688326 | 8 | 3767623 | 0.706 | T | C | -0.036 | 0.004 | -0.015 | 0.022 |  |
| rs2724961 | 8 | 4560227 | 0.468 | T | C | -0.046 | 0.004 | -0.005 | 0.020 |  |
| rs4875424 | 8 | 4831685 | 0.359 | T | C | -0.033 | 0.004 | 0.008 | 0.020 |  |
| rs6185 | 8 | 25280800 | 0.730 | C | G | -0.030 | 0.004 | -0.010 | 0.023 | Menopause (age at onset), heel bone mineral density |
| rs13278754 | 8 | 34902952 | 0.283 | C | G | -0.025 | 0.004 | 0.003 | 0.022 |  |
| rs4487799 | 8 | 53163528 | 0.303 | A | T | 0.024 | 0.004 | -0.044 | 0.022 |  |
| rs16918378 | 8 | 53877882 | 0.877 | T | C | 0.048 | 0.006 | -0.006 | 0.030 |  |
| rs56409371 | 8 | 53934144 | 0.784 | A | G | -0.035 | 0.005 | 0.005 | 0.025 |  |
| rs1449543 | 8 | 76591987 | 0.456 | T | C | 0.022 | 0.004 | 0.036 | 0.019 |  |
| rs11786868 | 8 | 77653945 | 0.837 | C | G | 0.032 | 0.005 | -0.006 | 0.026 | Menopause (age at onset) |
| rs10094506 | 8 | 78116203 | 0.281 | T | C | -0.045 | 0.004 | 0.000 | 0.022 |  |
| rs35485457 | 8 | 78679087 | 0.312 | T | G | -0.036 | 0.004 | -0.020 | 0.022 |  |
| rs7465046 | 8 | 87319950 | 0.232 | T | C | -0.041 | 0.005 | 0.003 | 0.024 |  |
| rs2441873 | 8 | 105329549 | 0.411 | T | G | 0.024 | 0.004 | 0.006 | 0.020 |  |
| rs7826872 | 8 | 132071766 | 0.447 | T | C | 0.026 | 0.004 | -0.018 | 0.016 |  |
| rs2542420 | 8 | 140645701 | 0.538 | C | G | 0.033 | 0.004 | 0.060 | 0.020 |  |
| rs552491 | 9 | 1711210 | 0.638 | A | G | -0.029 | 0.004 | -0.027 | 0.020 |  |
| rs913588 | 9 | 7174673 | 0.503 | A | G | -0.034 | 0.004 | 0.042 | 0.015 |  |
| rs10959016 | 9 | 10283451 | 0.206 | A | G | -0.031 | 0.005 | -0.009 | 0.025 |  |
| rs10959552 | 9 | 11130009 | 0.885 | A | G | -0.037 | 0.006 | 0.039 | 0.030 |  |
| rs1601615 | 9 | 11813745 | 0.396 | T | C | -0.028 | 0.004 | -0.040 | 0.020 |  |
| rs7849973 | 9 | 22819576 | 0.655 | C | G | 0.024 | 0.004 | -0.002 | 0.021 | Autism spectrum disorder, attention deficit-hyperactivity disorder, bipolar disorder, major depressive disorder, and schizophrenia (combined) |
| rs1329767 | 9 | 73798371 | 0.349 | A | C | -0.029 | 0.004 | -0.002 | 0.017 |  |
| rs2604265 | 9 | 76905178 | 0.266 | A | G | 0.039 | 0.004 | -0.023 | 0.022 |  |
| rs35436838 | 9 | 77273910 | 0.958 | T | G | -0.068 | 0.011 | -0.087 | 0.056 | Height |
| rs2378100 | 9 | 80513323 | 0.570 | T | C | -0.024 | 0.004 | -0.008 | 0.020 |  |
| rs4877387 | 9 | 81679875 | 0.282 | T | C | 0.024 | 0.004 | 0.001 | 0.022 | Amyotrophic lateral sclerosis in C9orf72 mutation positive individuals |
| rs11534296 | 9 | 83282402 | 0.273 | A | G | -0.037 | 0.004 | 0.013 | 0.022 |  |
| rs7853970 | 9 | 86715566 | 0.464 | T | C | 0.045 | 0.004 | -0.010 | 0.020 | Age of smoking initiation (MTAG) |
| rs13283567 | 9 | 86764996 | 0.152 | T | C | -0.044 | 0.006 | -0.031 | 0.029 |  |
| rs1571536 | 9 | 92215638 | 0.485 | T | C | 0.033 | 0.004 | -0.021 | 0.019 |  |
| rs9330454 | 9 | 92515514 | 0.430 | A | G | -0.031 | 0.004 | 0.033 | 0.024 |  |
| rs10992769 | 9 | 96276910 | 0.709 | C | G | 0.029 | 0.004 | -0.029 | 0.021 |  |
| rs10156597 | 9 | 108941509 | 0.677 | A | T | 0.102 | 0.004 | 0.005 | 0.021 | Menopause (age at onset) |
| rs56927240 | 9 | 109148074 | 0.201 | T | C | -0.057 | 0.005 | 0.009 | 0.024 |  |
| rs10978641 | 9 | 109554196 | 0.746 | A | T | -0.032 | 0.005 | -0.008 | 0.025 |  |
| rs11792861 | 9 | 111809295 | 0.709 | A | C | 0.032 | 0.004 | -0.007 | 0.017 |  |
| rs7852169 | 9 | 114318394 | 0.912 | C | G | -0.097 | 0.007 | -0.045 | 0.035 | Menopause (age at onset) |
| rs2780243 | 9 | 120730928 | 0.565 | T | C | -0.023 | 0.004 | -0.025 | 0.020 |  |
| rs4836984 | 9 | 127405632 | 0.493 | T | C | 0.034 | 0.004 | -0.029 | 0.016 |  |
| rs467379 | 9 | 136905474 | 0.307 | T | C | 0.024 | 0.004 | -0.014 | 0.022 |  |
| rs7907759 | 10 | 1730008 | 0.471 | A | G | 0.041 | 0.004 | 0.021 | 0.020 |  |
| rs7912468 | 10 | 2697434 | 0.578 | T | C | -0.024 | 0.004 | -0.017 | 0.020 |  |
| rs1885740 | 10 | 10251910 | 0.281 | A | G | -0.026 | 0.005 | -0.038 | 0.031 |  |
| rs10906395 | 10 | 13541008 | 0.611 | T | C | -0.023 | 0.004 | 0.000 | 0.020 |  |
| rs61846901 | 10 | 51056858 | 0.311 | T | C | -0.026 | 0.004 | 0.032 | 0.022 |  |
| rs6415872 | 10 | 63660689 | 0.493 | A | G | 0.024 | 0.004 | -0.013 | 0.020 |  |
| rs7072571 | 10 | 71380093 | 0.784 | A | G | 0.031 | 0.006 | 0.008 | 0.047 |  |
| rs4746113 | 10 | 74071178 | 0.309 | A | G | -0.024 | 0.004 | -0.011 | 0.023 |  |
| rs77532868 | 10 | 88081438 | 0.045 | T | C | 0.057 | 0.010 | 0.035 | 0.048 |  |
| rs1172955 | 10 | 97877320 | 0.700 | A | T | -0.044 | 0.004 | -0.018 | 0.021 |  |
| rs72842141 | 10 | 102686073 | 0.943 | A | T | -0.062 | 0.009 | -0.001 | 0.043 |  |
| rs59543819 | 10 | 103754188 | 0.705 | T | C | -0.025 | 0.004 | -0.033 | 0.021 |  |
| rs2066323 | 10 | 104871361 | 0.602 | A | G | -0.024 | 0.004 | -0.035 | 0.016 |  |
| rs10885077 | 10 | 112759731 | 0.255 | T | G | 0.024 | 0.004 | 0.006 | 0.021 |  |
| rs4751614 | 10 | 118696266 | 0.766 | A | T | 0.030 | 0.005 | 0.035 | 0.023 | Age of smoking initiation (MTAG) |
| rs10400136 | 10 | 120833948 | 0.563 | A | G | -0.026 | 0.004 | -0.046 | 0.021 |  |
| rs73435048 | 10 | 121154531 | 0.067 | A | G | -0.044 | 0.008 | -0.030 | 0.043 |  |
| rs12571664 | 10 | 121708929 | 0.800 | T | C | 0.037 | 0.005 | -0.065 | 0.024 |  |
| rs7077302 | 10 | 123676662 | 0.085 | C | G | 0.050 | 0.007 | 0.015 | 0.036 |  |
| rs9422857 | 10 | 126861278 | 0.506 | C | G | -0.028 | 0.004 | 0.010 | 0.020 |  |
| rs4576738 | 10 | 134294398 | 0.446 | A | G | 0.027 | 0.004 | 0.000 | 0.022 |  |
| rs3782120 | 11 | 206089 | 0.258 | A | G | 0.033 | 0.004 | -0.049 | 0.022 |  |
| rs16937956 | 11 | 8404501 | 0.638 | A | G | -0.038 | 0.004 | -0.001 | 0.018 | Body mass index |
| rs10832021 | 11 | 13324530 | 0.710 | A | G | -0.047 | 0.004 | -0.015 | 0.022 |  |
| rs4359170 | 11 | 16596152 | 0.673 | A | T | 0.028 | 0.004 | -0.006 | 0.017 |  |
| rs1032682 | 11 | 22791324 | 0.407 | T | C | 0.021 | 0.004 | 0.029 | 0.020 |  |
| rs16917237 | 11 | 27702383 | 0.209 | T | G | 0.039 | 0.005 | -0.005 | 0.024 | Body mass index |
| rs11606190 | 11 | 28033473 | 0.146 | A | G | 0.041 | 0.006 | -0.022 | 0.028 |  |
| rs6484408 | 11 | 28899164 | 0.287 | A | G | -0.025 | 0.004 | -0.017 | 0.022 |  |
| rs11031040 | 11 | 30317733 | 0.838 | T | G | -0.040 | 0.005 | 0.028 | 0.026 |  |
| rs1023955 | 11 | 43608835 | 0.398 | T | G | -0.028 | 0.004 | -0.001 | 0.020 |  |
| rs970179 | 11 | 45433845 | 0.472 | A | G | 0.021 | 0.004 | 0.011 | 0.019 |  |
| rs953230 | 11 | 46064974 | 0.708 | A | G | 0.033 | 0.004 | 0.013 | 0.022 |  |
| rs68002803 | 11 | 46539110 | 0.677 | T | C | 0.027 | 0.004 | 0.026 | 0.022 |  |
| rs10897450 | 11 | 63593219 | 0.530 | C | G | 0.023 | 0.004 | 0.006 | 0.020 |  |
| rs10750766 | 11 | 65473798 | 0.709 | A | C | -0.028 | 0.004 | 0.029 | 0.017 | Heel bone mineral density |
| rs7115444 | 11 | 77555824 | 0.209 | T | C | 0.033 | 0.005 | -0.004 | 0.024 |  |
| rs4945266 | 11 | 78027488 | 0.839 | A | G | -0.045 | 0.005 | -0.044 | 0.021 |  |
| rs4402316 | 11 | 84780098 | 0.241 | C | G | 0.031 | 0.005 | 0.022 | 0.026 |  |
| rs7108556 | 11 | 86716236 | 0.779 | T | C | 0.031 | 0.005 | -0.018 | 0.023 |  |
| rs113557523 | 11 | 94085099 | 0.086 | T | C | -0.045 | 0.007 | 0.000 | 0.036 |  |
| rs6590889 | 11 | 101438191 | 0.340 | T | C | -0.044 | 0.004 | -0.016 | 0.021 |  |
| rs17564430 | 11 | 115043574 | 0.747 | T | G | -0.035 | 0.004 | -0.042 | 0.022 |  |
| rs1815811 | 11 | 119059404 | 0.536 | A | G | -0.027 | 0.004 | -0.048 | 0.020 |  |
| rs7114175 | 11 | 122813983 | 0.496 | A | T | -0.060 | 0.004 | -0.008 | 0.019 |  |
| rs77530428 | 12 | 17126283 | 0.983 | A | G | -0.124 | 0.017 | -0.049 | 0.089 |  |
| rs10842343 | 12 | 24579079 | 0.604 | A | T | -0.026 | 0.004 | -0.023 | 0.017 |  |
| rs7971408 | 12 | 47876942 | 0.106 | T | C | 0.045 | 0.006 | 0.003 | 0.031 |  |
| rs1054442 | 12 | 49389320 | 0.625 | A | C | 0.036 | 0.004 | 0.018 | 0.021 | Intelligence, educational attainment |
| rs7132908 | 12 | 50263148 | 0.388 | A | G | -0.042 | 0.004 | -0.004 | 0.021 | Body mass index, alcohol consumption, waist circumference |
| rs1131017 | 12 | 56435929 | 0.417 | C | G | 0.023 | 0.004 | -0.073 | 0.016 | Cognitive performance (MTAG) |
| rs1148006 | 12 | 75978358 | 0.244 | A | G | -0.026 | 0.004 | 0.012 | 0.019 |  |
| rs7979001 | 12 | 97506357 | 0.508 | A | G | 0.022 | 0.004 | 0.017 | 0.016 |  |
| rs3764002 | 12 | 108618630 | 0.265 | T | C | -0.030 | 0.005 | 0.001 | 0.024 | Waist-to-hip ratio adjusted for BMI (additive genetic model) |
| rs11065822 | 12 | 111600134 | 0.373 | T | G | 0.026 | 0.004 | 0.054 | 0.022 |  |
| rs474463 | 12 | 115107376 | 0.219 | T | C | -0.029 | 0.005 | -0.014 | 0.022 |  |
| rs660549 | 12 | 121300988 | 0.567 | T | C | -0.021 | 0.004 | -0.003 | 0.019 | Educational attainment (MTAG) |
| rs9548873 | 13 | 40238492 | 0.663 | T | C | -0.031 | 0.004 | 0.098 | 0.016 |  |
| rs73187215 | 13 | 42646769 | 0.903 | A | G | -0.038 | 0.007 | -0.033 | 0.027 |  |
| rs9568123 | 13 | 49475780 | 0.848 | A | G | -0.029 | 0.005 | -0.023 | 0.027 |  |
| rs4886140 | 13 | 59833519 | 0.333 | A | G | 0.028 | 0.004 | 0.008 | 0.021 | Insomnia symptoms (never/rarely vs. usually) |
| rs1925047 | 13 | 74600274 | 0.321 | A | C | -0.034 | 0.004 | -0.012 | 0.021 |  |
| rs11619721 | 13 | 112082513 | 0.084 | T | G | -0.041 | 0.007 | -0.051 | 0.040 |  |
| rs9522262 | 13 | 112186283 | 0.492 | C | G | 0.041 | 0.004 | 0.049 | 0.021 |  |
| rs74499585 | 13 | 112285043 | 0.087 | A | G | 0.058 | 0.008 | 0.016 | 0.063 |  |
| rs10136330 | 14 | 30514335 | 0.043 | T | C | -0.058 | 0.010 | 0.055 | 0.049 |  |
| rs10138913 | 14 | 60943106 | 0.306 | T | C | 0.056 | 0.004 | -0.045 | 0.021 |  |
| rs10143972 | 14 | 93850179 | 0.804 | T | C | -0.039 | 0.005 | 0.016 | 0.026 |  |
| rs10145469 | 14 | 97769834 | 0.051 | A | C | -0.061 | 0.009 | -0.038 | 0.047 | Menopause (age at onset) |
| rs941520 | 14 | 99709702 | 0.494 | A | C | -0.022 | 0.004 | -0.020 | 0.020 | Adventurousness |
| rs12894936 | 14 | 100846991 | 0.294 | T | C | -0.052 | 0.004 | -0.037 | 0.023 |  |
| rs6575806 | 14 | 101353211 | 0.739 | A | C | -0.033 | 0.006 | 0.012 | 0.038 |  |
| rs79084266 | 14 | 101367407 | 0.881 | C | G | -0.040 | 0.007 | 0.008 | 0.042 |  |
| rs7178532 | 15 | 23794517 | 0.685 | A | G | 0.044 | 0.004 | 0.023 | 0.021 |  |
| rs4778356 | 15 | 24183428 | 0.865 | A | G | 0.039 | 0.006 | 0.009 | 0.034 |  |
| rs8040272 | 15 | 24824016 | 0.868 | A | G | 0.044 | 0.006 | 0.015 | 0.033 |  |
| rs34513772 | 15 | 40608820 | 0.670 | T | C | 0.024 | 0.004 | -0.002 | 0.023 |  |
| rs1435753 | 15 | 47925066 | 0.647 | T | C | -0.028 | 0.004 | -0.057 | 0.020 |  |
| rs28757192 | 15 | 51507610 | 0.034 | T | C | -0.063 | 0.011 | 0.055 | 0.043 |  |
| rs11852771 | 15 | 54364552 | 0.385 | A | G | 0.024 | 0.004 | -0.020 | 0.020 |  |
| rs3743266 | 15 | 60781513 | 0.668 | T | C | 0.042 | 0.004 | -0.034 | 0.021 | Height |
| rs72756954 | 15 | 64537300 | 0.938 | C | G | 0.058 | 0.008 | -0.050 | 0.041 |  |
| rs10153031 | 15 | 67987293 | 0.406 | T | G | 0.040 | 0.004 | 0.017 | 0.020 |  |
| rs5742915 | 15 | 74336633 | 0.546 | T | C | -0.023 | 0.004 | 0.023 | 0.021 | Height |
| rs1971554 | 15 | 83406228 | 0.265 | T | C | 0.032 | 0.004 | -0.007 | 0.019 |  |
| rs12915845 | 15 | 89042467 | 0.424 | T | C | -0.040 | 0.004 | 0.008 | 0.015 |  |
| rs758747 | 16 | 3627358 | 0.276 | T | C | -0.027 | 0.004 | -0.041 | 0.024 | Body mass index |
| rs1704528 | 16 | 14388750 | 0.662 | T | C | -0.051 | 0.004 | -0.029 | 0.021 | Male-pattern baldness |
| rs153793 | 16 | 15542199 | 0.520 | A | G | -0.023 | 0.004 | 0.009 | 0.020 |  |
| rs112991346 | 16 | 19967668 | 0.858 | T | C | -0.045 | 0.006 | -0.010 | 0.028 |  |
| rs4780885 | 16 | 20380004 | 0.493 | C | G | 0.026 | 0.004 | 0.012 | 0.020 | Body mass index |
| rs113388806 | 16 | 24804954 | 0.958 | A | T | -0.062 | 0.010 | 0.041 | 0.055 |  |
| rs8051833 | 16 | 29896390 | 0.342 | A | G | -0.040 | 0.004 | 0.034 | 0.022 |  |
| rs3809624 | 16 | 30102802 | 0.677 | T | C | -0.030 | 0.004 | 0.039 | 0.019 |  |
| rs143461173 | 16 | 52283158 | 0.806 | A | G | 0.029 | 0.005 | 0.010 | 0.025 |  |
| rs9972653 | 16 | 53814363 | 0.400 | T | G | -0.051 | 0.004 | -0.003 | 0.016 | Body fat mass, Lean body mass, Heel bone mineral density |
| rs7359336 | 16 | 69733460 | 0.579 | A | G | -0.053 | 0.004 | -0.008 | 0.020 |  |
| rs4448948 | 16 | 72569236 | 0.929 | A | T | -0.041 | 0.008 | -0.059 | 0.038 |  |
| rs112752732 | 17 | 1942577 | 0.046 | C | G | 0.057 | 0.010 | -0.002 | 0.054 |  |
| rs142643995 | 17 | 2017993 | 0.030 | T | C | 0.065 | 0.012 | 0.000 | 0.057 |  |
| rs12603280 | 17 | 6034754 | 0.244 | A | G | -0.037 | 0.005 | 0.004 | 0.023 |  |
| rs55680968 | 17 | 7774047 | 0.928 | A | G | -0.046 | 0.008 | -0.022 | 0.038 |  |
| rs59246405 | 17 | 43123625 | 0.405 | T | C | 0.027 | 0.004 | -0.005 | 0.020 |  |
| rs11079810 | 17 | 46227846 | 0.105 | T | C | 0.036 | 0.006 | -0.027 | 0.025 | Lipid traits (pleiotropy) (HIPO component 1) |
| rs9635759 | 17 | 49613785 | 0.307 | A | G | 0.059 | 0.004 | 0.018 | 0.022 | Menopause (age at onset) |
| rs2787487 | 17 | 53209382 | 0.604 | C | G | 0.031 | 0.004 | 0.004 | 0.016 |  |
| rs7218751 | 17 | 77796437 | 0.811 | A | G | 0.033 | 0.005 | -0.006 | 0.025 |  |
| rs59652033 | 17 | 77951023 | 0.348 | T | C | -0.026 | 0.004 | 0.049 | 0.025 |  |
| rs66508321 | 17 | 78739672 | 0.324 | A | G | -0.030 | 0.004 | 0.000 | 0.021 |  |
| rs2659007 | 17 | 79217478 | 0.465 | A | G | -0.031 | 0.004 | -0.033 | 0.020 |  |
| rs12937034 | 17 | 79446015 | 0.614 | A | G | -0.025 | 0.004 | 0.005 | 0.022 |  |
| rs11873906 | 18 | 3813464 | 0.720 | A | G | -0.051 | 0.004 | 0.000 | 0.022 | Height |
| rs8087304 | 18 | 31765736 | 0.482 | A | T | 0.022 | 0.004 | -0.007 | 0.019 |  |
| rs1512238 | 18 | 44748467 | 0.421 | A | G | -0.054 | 0.004 | 0.002 | 0.020 |  |
| rs7239114 | 18 | 45921214 | 0.540 | A | G | -0.022 | 0.004 | -0.010 | 0.016 | Body mass index |
| rs3746037 | 19 | 1828948 | 0.787 | A | C | 0.036 | 0.005 | 0.004 | 0.027 |  |
| rs169080 | 19 | 4980864 | 0.354 | T | C | -0.026 | 0.004 | -0.024 | 0.017 | Smoking initiation, SLE |
| rs484353 | 19 | 7891767 | 0.539 | A | G | 0.032 | 0.004 | 0.002 | 0.015 |  |
| rs4804117 | 19 | 9984509 | 0.557 | T | G | 0.046 | 0.004 | 0.005 | 0.020 |  |
| rs10422323 | 19 | 13104027 | 0.121 | A | G | 0.037 | 0.006 | -0.030 | 0.035 |  |
| rs12460047 | 19 | 18346228 | 0.283 | A | G | -0.028 | 0.004 | -0.047 | 0.018 |  |
| rs11668587 | 19 | 18829770 | 0.665 | A | G | -0.033 | 0.004 | 0.020 | 0.017 |  |
| rs56367141 | 19 | 31051857 | 0.131 | A | C | -0.040 | 0.006 | -0.042 | 0.032 |  |
| rs29941 | 19 | 34309532 | 0.323 | A | G | 0.028 | 0.004 | 0.006 | 0.016 | Body mass index |
| rs4804025 | 19 | 47609223 | 0.704 | A | G | -0.041 | 0.004 | -0.002 | 0.017 |  |
| rs2548458 | 19 | 49209325 | 0.506 | T | C | 0.021 | 0.004 | 0.002 | 0.017 |  |
| rs4801809 | 19 | 50334895 | 0.096 | T | C | -0.044 | 0.007 | -0.030 | 0.045 |  |
| rs2889128 | 19 | 58973929 | 0.456 | A | C | 0.022 | 0.004 | 0.010 | 0.020 |  |
| rs852061 | 20 | 17109159 | 0.365 | A | C | -0.037 | 0.004 | 0.003 | 0.020 |  |
| rs1535252 | 20 | 19682834 | 0.455 | T | C | -0.025 | 0.004 | 0.005 | 0.016 |  |
| rs111558392 | 20 | 20348962 | 0.153 | T | C | -0.040 | 0.006 | 0.000 | 0.032 |  |
| rs4813429 | 20 | 21485806 | 0.831 | T | C | 0.031 | 0.005 | -0.007 | 0.027 |  |
| rs1737894 | 20 | 31054702 | 0.600 | C | G | 0.022 | 0.004 | -0.002 | 0.016 | Cigarettes smoked per day (MTAG) |
| rs2295094 | 20 | 33447915 | 0.159 | A | G | 0.036 | 0.005 | 0.016 | 0.026 | Neurociticism |
| rs36093651 | 20 | 37287102 | 0.238 | T | C | 0.037 | 0.005 | 0.009 | 0.023 |  |
| rs2425674 | 20 | 43529461 | 0.439 | C | G | -0.022 | 0.004 | -0.020 | 0.016 |  |
| rs3746619 | 20 | 54823805 | 0.086 | A | C | 0.048 | 0.007 | -0.015 | 0.035 |  |
| rs13043968 | 20 | 54830983 | 0.111 | A | C | -0.041 | 0.006 | 0.012 | 0.032 |  |
| rs443252 | 20 | 62799680 | 0.050 | T | C | 0.059 | 0.009 | -0.064 | 0.058 |  |
| rs62229372 | 21 | 37692507 | 0.124 | T | C | 0.051 | 0.006 | -0.037 | 0.037 | blood pressure |
| rs117143374 | 21 | 40555561 | 0.864 | T | C | -0.050 | 0.006 | -0.069 | 0.028 |  |
| rs151680 | 22 | 22273242 | 0.524 | T | C | 0.028 | 0.004 | -0.051 | 0.020 |  |
| rs5753377 | 22 | 31293700 | 0.287 | A | G | -0.031 | 0.004 | -0.042 | 0.022 |  |
| rs9614460 | 22 | 45745229 | 0.678 | T | G | -0.025 | 0.004 | 0.021 | 0.017 |  |
| rs8136272 | 22 | 49678782 | 0.728 | A | T | 0.040 | 0.004 | -0.027 | 0.022 |  |
| A1: effect allele; A2: the other allele; Chr: chromosome. | | | | | | | | | | |

| **Supplementary Table 2. The characteristic of age at natural menopause associated index SNPs, their effect sizes with exposure and outcome, as well as their associations with potential confounders.** | | | | | | | | | | |
| --- | --- | --- | --- | --- | --- | --- | --- | --- | --- | --- |
| SNP | Chr | Position | Allele frequency | A1 | A2 | Exposure | | Outcome | | Confounder |
|  |  |  |  |  |  | beta | se | beta | se |  |
| rs12142240 | 1 | 46747301 | 0.32 | C | T | 0.130 | 0.020 | 0.038 | 0.021 |  |
| rs1411478 | 1 | 180962282 | 0.59 | G | A | 0.130 | 0.020 | 0.019 | 0.020 | Progressive supranuclear palsy |
| rs2236918 | 1 | 242017826 | 0.55 | G | C | 0.150 | 0.020 | -0.004 | 0.021 |  |
| rs4246511 | 1 | 39380385 | 0.29 | T | C | 0.020 | 0.020 | -0.066 | 0.022 |  |
| rs1800932 | 2 | 48018081 | 0.19 | G | A | 0.170 | 0.030 | 0.023 | 0.025 |  |
| rs704795 | 2 | 27716494 | 0.6 | G | A | 0.160 | 0.020 | -0.023 | 0.016 |  |
| rs930036 | 2 | 171941018 | 0.62 | G | A | 0.190 | 0.020 | 0.012 | 0.016 |  |
| rs16858210 | 3 | 183624010 | 0.25 | A | G | 0.140 | 0.020 | 0.009 | 0.022 |  |
| rs4693089 | 4 | 84373622 | 0.49 | G | A | 0.200 | 0.020 | -0.011 | 0.019 | Menarche (age at onset) |
| rs6856693 | 4 | 185748806 | 0.42 | G | A | 0.160 | 0.020 | -0.039 | 0.021 |  |
| rs11738223 | 5 | 171934492 | 0.32 | G | A | 0.120 | 0.020 | -0.008 | 0.021 |  |
| rs2241584 | 5 | 175956177 | 0.62 | G | A | 0.140 | 0.020 | -0.008 | 0.020 |  |
| rs365132 | 5 | 176378574 | 0.49 | T | G | 0.240 | 0.020 | 0.006 | 0.019 | Menarche (age at onset) |
| rs427394 | 5 | 6745875 | 0.59 | A | G | 0.130 | 0.020 | -0.011 | 0.020 |  |
| rs12196873 | 6 | 111598058 | 0.15 | C | A | 0.160 | 0.030 | -0.010 | 0.022 |  |
| rs2230365 | 6 | 31525448 | 0.16 | T | C | 0.170 | 0.030 | 0.216 | 0.021 |  |
| rs6899676 | 6 | 10895260 | 0.2 | G | A | 0.230 | 0.030 | -0.011 | 0.025 | Menarche (age at onset) |
| rs707938 | 6 | 31729359 | 0.68 | A | G | 0.170 | 0.020 | 0.059 | 0.017 | Menarche (age at onset), General cognitive ability, Cognitive performance |
| rs9393800 | 6 | 10951737 | 0.73 | A | G | 0.170 | 0.020 | 0.010 | 0.022 | Menarche (age at onset) |
| rs10957156 | 8 | 61629401 | 0.24 | G | A | 0.140 | 0.020 | 0.030 | 0.019 |  |
| rs2720044 | 8 | 37980587 | 0.16 | C | A | 0.290 | 0.030 | -0.071 | 0.034 |  |
| rs4879656 | 9 | 33012382 | 0.63 | C | A | 0.120 | 0.020 | 0.006 | 0.020 |  |
| rs10905065 | 10 | 5769827 | 0.39 | G | A | 0.110 | 0.020 | -0.011 | 0.020 |  |
| rs10734411 | 11 | 32541784 | 0.53 | G | A | 0.120 | 0.020 | -0.010 | 0.020 |  |
| rs11031006 | 11 | 30226528 | 0.15 | A | G | 0.220 | 0.030 | -0.042 | 0.028 | Number of twin births, Polycystic ovary syndrome, Length of menstrual cycle |
| rs6484478 | 11 | 30306440 | 0.26 | A | G | 0.100 | 0.020 | -0.010 | 0.023 |  |
| rs1183272 | 12 | 66735421 | 0.55 | T | C | 0.070 | 0.020 | 0.026 | 0.019 |  |
| rs12824058 | 12 | 130804334 | 0.57 | A | G | 0.140 | 0.020 | 0.008 | 0.020 |  |
| rs1727326 | 12 | 123600086 | 0.85 | G | C | 0.190 | 0.030 | -0.011 | 0.024 |  |
| rs2277339 | 12 | 57146069 | 0.9 | T | G | 0.310 | 0.030 | 0.053 | 0.035 | Red cell distribution width, Waist-hip ratio, Fat-free mass, Mean corpuscular volume, Height |
| rs3741604 | 12 | 66696410 | 0.48 | C | T | 0.090 | 0.020 | -0.013 | 0.019 |  |
| rs551087 | 12 | 121209193 | 0.71 | A | G | 0.130 | 0.020 | -0.040 | 0.021 |  |
| rs7397861 | 12 | 66814466 | 0.36 | C | G | 0.100 | 0.020 | 0.020 | 0.020 |  |
| rs4886238 | 13 | 61113739 | 0.34 | A | G | 0.180 | 0.020 | -0.016 | 0.021 |  |
| rs1713460 | 14 | 20933615 | 0.7 | A | G | 0.140 | 0.020 | 0.020 | 0.024 |  |
| rs1054875 | 15 | 89879126 | 0.6 | A | T | 0.190 | 0.020 | -0.012 | 0.016 |  |
| rs9796 | 15 | 41271447 | 0.54 | A | T | 0.130 | 0.020 | 0.030 | 0.020 |  |
| rs10852344 | 16 | 12016919 | 0.41 | C | T | 0.160 | 0.020 | -0.005 | 0.020 |  |
| rs12599106 | 16 | 34498025 | 0.49 | T | A | 0.120 | 0.020 | -0.009 | 0.021 |  |
| rs9039 | 16 | 9205363 | 0.72 | T | C | 0.120 | 0.020 | 0.036 | 0.022 |  |
| rs1799949 | 17 | 41245466 | 0.32 | A | G | 0.140 | 0.020 | 0.010 | 0.021 | Menarche (age at onset) |
| rs2941505 | 17 | 37832704 | 0.68 | G | A | 0.130 | 0.020 | 0.078 | 0.017 |  |
| rs8070740 | 17 | 5331896 | 0.24 | G | A | 0.150 | 0.020 | 0.026 | 0.023 |  |
| rs11668344 | 19 | 55833664 | 0.64 | A | G | 0.410 | 0.020 | 0.028 | 0.016 | Menarche (age at onset) |
| rs12461110 | 19 | 56320663 | 0.65 | G | A | 0.170 | 0.020 | 0.032 | 0.021 |  |
| rs2547274 | 19 | 56310228 | 0.09 | C | G | 0.280 | 0.040 | -0.044 | 0.036 |  |
| rs349306 | 19 | 950694 | 0.87 | A | G | 0.230 | 0.040 | -0.012 | 0.047 |  |
| rs7259376 | 19 | 22507705 | 0.54 | G | A | 0.110 | 0.020 | -0.022 | 0.019 |  |
| rs13040088 | 20 | 61549202 | 0.79 | A | G | 0.160 | 0.020 | 0.014 | 0.024 |  |
| rs16991615 | 20 | 5948227 | 0.07 | A | G | 0.880 | 0.040 | 0.004 | 0.045 | Breast cancer, Uterine fibroids |
| rs2236553 | 20 | 61289743 | 0.76 | T | C | 0.160 | 0.030 | 0.005 | 0.029 |  |
| rs451417 | 20 | 5941999 | 0.88 | C | A | 0.200 | 0.030 | 0.029 | 0.030 |  |
| rs5762534 | 22 | 28633571 | 0.16 | C | T | 0.160 | 0.030 | 0.006 | 0.027 | Cleft lip with or without cleft palate (maternal periconceptional vitamin use interaction), Menarche (age at onset) |
| rs763121 | 22 | 38879940 | 0.64 | A | G | 0.160 | 0.020 | -0.044 | 0.017 |  |
| A1: effect allele; A2: the other allele; Chr: chromosome. | | | | | | | | | | |

| **Supplementary Table 3. The characteristic of age at first birth associated index SNPs, their effect sizes with exposure and outcome.** | | | | | | | | | |
| --- | --- | --- | --- | --- | --- | --- | --- | --- | --- |
| SNP | Chr | Position | Allele frequency | A1 | A2 | Exposure | | Outcome | |
|  |  |  |  |  |  | beta | se | beta | se |
| rs10908557 | 1 | 153927052 | 0.695 | C | G | 0.091 | 0.015 | 0.026 | 0.021 |
| rs1160544 | 2 | 100832218 | 0.395 | A | C | -0.082 | 0.014 | 0.082 | 0.016 |
| rs2777888 | 3 | 49898000 | 0.507 | A | G | 0.106 | 0.013 | 0.008 | 0.015 |
| rs10056247 | 5 | 133898136 | 0.289 | T | C | 0.082 | 0.016 | -0.040 | 0.017 |
| rs6885307 | 5 | 45094503 | 0.799 | A | C | -0.107 | 0.017 | 0.016 | 0.025 |
| rs2347867 | 6 | 152229850 | 0.649 | A | G | 0.091 | 0.015 | -0.020 | 0.021 |
| rs10953766 | 7 | 114313218 | 0.429 | A | G | 0.087 | 0.014 | -0.018 | 0.020 |
| rs2721195 | 8 | 145677011 | 0.469 | T | C | -0.073 | 0.016 | 0.027 | 0.021 |
| rs293566 | 20 | 31097877 | 0.650 | T | C | 0.081 | 0.015 | 0.037 | 0.021 |
| rs242997 | 22 | 34503059 | 0.613 | A | G | -0.084 | 0.014 | -0.010 | 0.020 |
| A1: effect allele; A2: the other allele; Chr: chromosome. | | | | | | | | | |
